# Prevalence and risk factors of latent tuberculosis infection among health care workers in Malaysia

**DOI:** 10.1186/1471-2334-11-19

**Published:** 2011-01-18

**Authors:** Shaharudin Rafiza, Krishna Gopal Rampal, Aris Tahir

**Affiliations:** 1 Institute for Medical Research, Environmental Health Research Centre, Occupational Health Unit, Jalan Pahang, 50588 Kuala Lumpur, Malaysia; 2 Universiti Kebangsaan Malaysia Medical Centre, Department of Community Health, Jalan Yaacob Latif, 56000 Kuala Lumpur, Malaysia; 3 Institute for Public Health, Jalan Bangsar, 50590 Kuala Lumpur, Malaysia

## Abstract

**Background:**

Health care workers are exposed to patients with tuberculosis and are at risk of nosocomial infection. The aim of this study was to determine the prevalence and factors associated with latent tuberculosis infection among health care workers in Malaysia and also to evaluate the agreement between Quantiferon TB Gold in tube test with Tuberculin Skin Test.

**Methods:**

A cross sectional study was conducted at four randomly selected hospitals in the Klang Valley from December 2008 to May 2009. Self administered questionnaire was used to obtain information on health care workers and possible risk factors. The response rate for this study was 90.8% with 954 respondents completed the questionnaire and were tested with Quantiferon TB Gold in tube for latent tuberculosis infection. Agreement between Quantiferon TB Gold in tube and Tuberculin Skin Test was assessed among 95 health care workers who consented to undergo both tests.

**Results:**

The overall prevalence of latent tuberculosis infection among health care workers was 10.6% (CI: 8.6%; 12.6%). Factors significantly associated with latent tuberculosis infection were aged 35 years and older [9.49 (CI: 2.22; 40.50)], history of living in the same house with close family members or friends who had active tuberculosis [8.69 (CI: 3.00; 25.18)], worked as a nurse [4.65 (CI: 1.10; 19.65)] and being male [3.70 (CI: 1.36; 10.02)]. Agreement between Quantiferon TB Gold in tube test and tuberculin skin test at cut-off points of 10 mm and 15 mm was 50.5% and 82.1% respectively. However, Kappa-agreement was poor for both cut-off points.

**Conclusion:**

The prevalence of latent tuberculosis infection in Malaysia was relatively low for an intermediate TB burden country. We could not comment on the occupational risk of latent tuberculosis infection among health care worker compared to the general population as there were no prevalence data available for latent tuberculosis infection in the general population. Kappa agreement between Quantiferon TB gold in-tube and tuberculin skin test was poor.

## Background

Tuberculosis (TB) in the era of HIV/AIDs, is the second most frequent cause of death due to an infectious agent [1]. Similar to other developing countries, TB is still a public health problem in Malaysia despite preventive and control measures taken. The incidence rate in Malaysia has been stagnant at around 58.7 to 65.6 per 100,000 populations in the last ten years. However, the absolute number of new cases has been increasing from about 15,000 new cases in 2002 up to 16,665 in 2006 [2]. As in other countries the re-emergence of this problem can be attributed to the high influx of foreign workers from high TB burden countries (legal and illegal), increasing number of HIV/AIDs patients, transmission within congregate settings, and emergence of multi drug resistant TB [3]. The higher number of patients seeking treatment at health facilities increases the exposure to health care workers. Based on the data collected by the Tuberculosis Information System (TBIS), Ministry of Health, Malaysia, it showed that the incidence rate of active TB disease among health care workers (HCWs) was higher compared to the general population in 2003 until 2006 with the reported incidence rate of 73.4-77.7 per 100,000 among HCWs and 60.3-62.6 per 100,000 in the general population respectively. There was an increasing trend in the number of HCWs diagnosed with active TB disease from 31 in 2002 to 123 in 2006 [2].

Individuals with latent tuberculosis infection (LTBI) are assumed to harbour viable tubercle bacilli in their body. These bacilli are dormant but have the potential to reactivate and cause disease. Previously, LTBI was diagnosed using tuberculin skin test (TST). TST measures the hypersensitivity response to purified protein derivative (PPD), which contains a mixture of antigens found in *Mycobacterium tuberculosis, M. bovis *and several other non-tubercular mycobacteria. However in the last decades, 2 new tests [interferon gamma releasing assays (IGRA)] have been developed. Quantiferon TB Gold in-tube (QFT TB Git) is one of the IGRA that is available commercially. It uses enzyme-linked immunosorbent assay (ELISA) to measure the production of interferon gamma by circulating T cells in whole blood against specific *Mycobacterium tuberculosis *antigens, namely overlapping peptides corresponding to ESAT-6 (early secreted antigenic target 6), CFP-10 (culture filtrate protein 10) and a portion of tuberculosis antigen TB7.7 (Rv2654). IGRA are more specific than TST as the antigens used are not shared by any of the BCG vaccines or by non-tubercular mycobacteria [4]. Results from a meta-analysis which reviewed the sensitivity between TST (cut-off point of 15 mm) and QFT TB Git using active tuberculosis as a surrogate for latent infection revealed a pooled sensitivity of 67% for QFT TB Git and 40% for TST [5]. The same study reported specificity of QFT as 96%, and only 87% for TST among healthy population considered at very low risk for LTBI.

As there is a paucity of data on the risk of tuberculosis among health care workers in Malaysia and there are no published study using any IGRA in testing for LTBI locally, a study was conducted to assess the prevalence and risk factors for latent TB infection among hospital workers in the Klang Valley. The agreement between TST and IGRA among a subpopulation of this study was also evaluated.

## Methods

### Sampling and Study Design

A cross sectional study was conducted from December 2008 to May 2009 among health care workers working in hospitals under the Ministry of Health in the Klang Valley, which was part of a bigger study aimed at identifying risk factors of LTBI among workers in the high and low risk groups. In total there were eight hospitals under the Ministry of Health in the area studied. In order to attain adequate number of respondents for this study, HCWs from four hospitals were required. Therefore, four hospitals were selected by simple random sampling. Workers were divided into high and low risk based on exposure to patients with active tuberculosis. The high risk group comprised of workers who are exposed to active TB patients or specimens regularly. They were from the medical ward, intensive care unit (ICU), emergency department (ED) and microbiology laboratory, whilst the low risk group were workers who are not in direct contact or not exposed to TB patients regularly. They comprised of workers from administration office and obstetric wards including labour room (LR). The workers involved in this study were nurses, medical assistants (MA), medical laboratory technicians (MLT) and office workers. Sample size calculation was based on a prevalence rate of 4% in the low risk group and an adjusted odds ratio of 2.48 among the high risk group based on another study in an intermediate TB burden country [6]. At α error of 0.05 and β error of 0.20 and 40% non response rate, the minimum sample size required was 525 in each group.

The respondents were selected randomly using probability proportionate to size. The formula used to calculate the sample for each hospital was number of workers for a given hospital over total number of workers from the four selected hospitals multiplied by number of samples required (525). Similar method of calculation was used to calculate number of samples according to each ward/department selected.

### Data collection

Self administered questionnaire were distributed to gather information on possible risk factors (sociodemographic, work history, BCG vaccination, co-morbid conditions, substance abuse, history of previous TB, and history of living in the same house with a family member or friend with active TB). In this study, a diagnosis of LTBI was made if the respondent was tested positive by Quantiferon TB Gold in-tube test (Cellestis Limited, Carnegie, Australia). The test was performed according to the manufacturer's instructions which included 2 stages: incubation of whole blood in the Nil Control, TB Antigen and Mitogen Control tubes for 16 to 24 hours at 37°C and harvesting the plasma from each tube, and measurement of interferon γ (IFN-γ) production in harvested plasma by enzyme-linked immunosorbent assay (ELISA) and results were calculated with QFT TB Git Analysis software. The IFN-γ values for tuberculosis antigens and mitogen were corrected for negative control (IFN-γ level of the Nil Control) for each respondent. The test was considered positive when the corrected TB Antigen stimulated plasma level was ≥ 0.35 IU/mL.

We also sought to determine the agreement between QFT Git with tuberculin skin test (TST) in 95 of the nurses who consented to be tested by both tests. TST was performed after blood for QFT Git has been taken. The test was administered by a trained nurse using the Mantoux method i.e. 0.1 ml of 2 T.U. of Tuberculin PPD RT 23 SSI was injected intradermally at the volar aspect of the forearm of respondents. The test was read 48 to 72 hours after application using the palpation method. Agreement and Kappa values were calculated for the two tests. Two cut off points for a positive TST (10 and 15 mm of induration) were evaluated.

### Data Analysis

All HCW who agreed to participate were included for the analysis. Analysis was done using SPSS, version 18 (SPSS Inc). Odds ratio and multiple logistic regression analysis were used to calculate crude and adjusted risk. The study protocol was approved by the Medical Research and Ethical Committee, Ministry of Health.

## Results

Of 1050 eligible health care workers, 954 (90.8%) answered the questionnaire and agreed to be tested for LTBI with Quantiferon TB Gold in-tube (QFT Git). Of the non respondents, 37 (38.5%) did not return the questionnaires and refused to be screened for LTBI, 52 (54.2%) agreed to answer the questionnaire only and 7 (7.3%) were screened for LTBI but did not return the questionnaire. All of the non respondents were from the low risk group except for one medical laboratory technician who was from the high risk group. Among the respondents, analysis was carried out among 953 respondents only as one respondent tested indeterminate by QFT Git and was excluded from this study. Majority of the respondents were females (88.0%), Malays (92.0%) and nurses (76.0%). This reflected the current work force in most Malaysian hospitals. The mean age of respondents was 29.2 years (range 19 to 56 years old; SD 7.8) and mean duration of employment in the Ministry of Health was 6.8 years (range less than 1 year to 38 years; SD 7.3). Almost all of the respondents (99.7%) had received BCG vaccination. Among those who received the vaccination, 90.7% received the last vaccination at entry to primary school (7 years old) or before the end of their primary school (12 years old).

In this study, the overall prevalence rate of LTBI among HCW was 10.6% (CI: 8.6%; 12.6%). Univariable analysis showed significantly high risk among those aged between 25 and 29 years old [1.89 (CI: 1.02; 3.50)] and 35 years and above [4.77 (CI: 2.57; 8.85)], ever married [1.69 (CI: 1.04; 2.74)], lived in the same house with close family members or friends who had active tuberculosis [6.58 (CI: 2.58; 16.76)] and had history of active TB previously [4.32 (CI: 1.06; 17.53)] (Table 1). Whilst, occupational factors including job category, workplace and risk group were not significantly associated with LTBI (Table 2). The only occupational factor found to be significantly associated with higher prevalence of LTBI was duration of employment 11 years or more [3.48 (CI: 1.57; 7.72)]. On multivariable analysis, the factors found to be significantly associated with LTBI were aged 35 and above [9.49 (CI: 2.22; 40.50)], history of living in the same house with close family members or friends who had active tuberculosis [8.69 (CI: 3.00; 25.18)], worked as a nurse [4.65 (CI: 1.10; 19.65)] and being male [3.70 (CI: 1.36; 10.02)].

**Table 1:** Prevalence of latent tuberculosis by sociodemographic characteristics and other risk factors.

Variable	Total no tested, N	No positive, n (%)	Crude OR	95% CI OR	Adjusted OR	95% CI OR
**Age group**						
≤ 24 years	293	16 (5.5)	1.00			
25 to 29 years	336	33 (9.8)	1.89*	1.02; 3.50	1.95	0.94; 4.05
30 to 34 years	148	14 (9.5)	1.81	0.86; 3.82	2.48	0.79; 7.79
≥ 35 years	176	38 (21.6)	4.77*	2.57; 8.85	9.49*	2.22; 40.50
**Sex**						
Female	839	87 (10.4)	1.00			
Male	114	14 (12.3)	1.21	0.66; 2.21	3.70*	1.36; 10.02
**Marital Status**						
Single	306	23 (7.5)	1.00			
Ever Married	647	78 (12.1)	1.69*	1.04;2.74	1.16	0.63; 2.14
**Race**						
Malays	877	92 (10.5)	1.00			
Non Malays	76	9 (11.8)	1.15	0.55; 2.38	1.14	0.51; 2.51
**Level of Education**						
High school	267	25 (9.4)	1.00			
College/Tertiary	686	76 (11.1)	1.21	0.75; 1.94	1.13	0.58; 2.19
**Income group**						
Low	33	2 (6.1)	1.00			
Middle	706	60 (8.5)	1.44	0.34; 6.16	0.99	0.17; 5.69
High	214	39 (18.2)	3.45	0.79; 15.04	1.38	0.18; 10.73
**Body Mass Index**						
Normal	421	45 (10.7)	1.00			
Underweight	173	21 (12.1)	1.15	0.66; 2.00	1.67	0.91; 3.06
Overweight/Obese	352	35 (9.9)	0.92	0.58; 1.47	0.75	0.45; 1.26
**Lived in same house friend/family with active TB**						
No	934	93 (10.0)	1.00			
Yes	19	8 (42.1)	6.58*	2.58; 16.76	8.69*	3.00; 25.18
**Co-morbid condition**						
No	928	96 (10.3)	1.00			
Yes	25	5 (20.0)	2.17	0.80; 5.90	1.09	0.36; 3.30
**Previous active TB**						
No	944	98 (10.4)	1.00			
Yes	9	3 (33.3)	4.32*	1.06; 17.53	1.41	0.10; 20.76
**Had Received TB treatment**						
No	938	97 (10.3)	1.00			
Yes	15	4 (26.7)	3.15	0.98; 10.09	1.92	0.20; 18.44
**Substance abuse**						
No	915	98 (10.7)	1.00			
Yes	38	3 (7.9)	0.72	0.22; 2.37	0.79	0.19; 3.27

**Table 2:** Prevalence of latent tuberculosis by occupational risk factors.

Variable	Total no tested, N	No positive, n (%)	Crude OR	95% CI OR	Adjusted OR	95% CI OR
**Duration of employment**						
<1 year	131	8 (6.1)	1.00			
1 to 5 years	448	38 (8.5)	1.42	0.65; 3.14	1.11	0.44; 2.78
6 to 10 years	168	17 (10.1)	1.73	0.72; 4.15	1.06	0.31; 3.63
≥ 11 years	206	38 (18.4)	3.48*	1.57; 7.72	0.56	0.11; 2.76
**Risk group**						
Low risk	430	45 (10.5)	1.00			
High risk	523	56 (10.7)	1.03	0.68; 1.55	1.79	0.02; 154.20
**Job category**						
Nurse	724	82 (11.3)	1.41	0.84; 2.38	4.65*	1.10; 19.65
Others	229	19 (8.3)	1.00			
**Workplace**						
Medical Ward	190	26 (13.7)	1.32	0.66; 2.65	0.25	0.00; 27.53
ICU	149	12 (8.1)	0.73	0.33; 1.64	0.16	0.00; 17.75
Emergency Dept	155	18 (11.6)	1.11	0.53; 2.32	0.21	0.00; 22.14
Obstetric	299	31 (10.4)	0.96	0.49; 1.88	0.24	0.05; 1.10
Microbiology Lab	29	0 (0)				
Administration	131	14 (10.7)	1.00			
**Clinical Base**						
Clinical	822	87 (10.6)	0.99	0.54; 1.80		
Non clinical	131	14 (10.7)	1.00			

**Figure 1:**
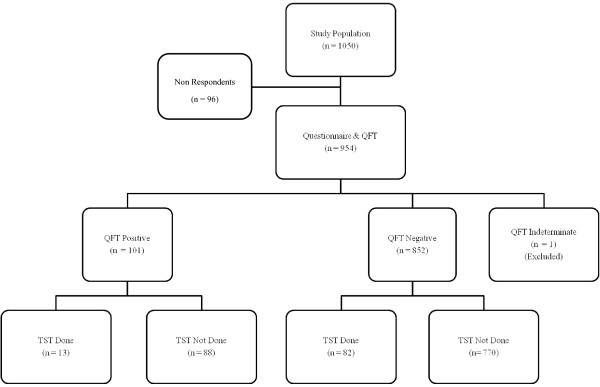
**Description of study population by Quantiferon TB Gold in tube and Tuberculin Skin Test**.

Only 95 respondents agreed to be tested by both QFT TB Git and TST as illustrated in Figure 1. Agreement between QFT TB gold in-tube and TST was assessed using cut-off points of 10 mm and 15 mm. For the cut off point of 10 mm for a positive TST, 37 (38.9%) were negative by both tests and 11 (11.6%) were positive by both tests. Whilst when 15 mm was used as the cut off point, 72 (75.8%) were negative by both tests and only 6 (6.3%) were positive by both tests. Overall agreement by 10 mm and 15 mm cut off point was 50.5% and 82.1% respectively. The κ agreement between QFT TB gold in-tube and TST was poor for both cut off points of 10 mm and 15 mm, at 0.12 and 0.31 respectively (Table 3).

**Table 3:** Agreement between QFT Gold In-tube and TST

Results	TST Cut off point, mm	
**TST/QFT Git**	**≥ 10 mm**	**≥ 15 mm**

Negative/Negative	37 (38.9%)	72 (75.8%)
Positive/Positive	11 (11.6%)	6 (6.3%)
Positive/Negative	45 (47.4%)	10 (10.5%)
Negative/Positive	2 (2.1%)	7 (7.4%)
Agreement (%)	48 (50.5%)	80 (82.1%)
Kappa	0.12	0.31

## Discussion

The prevalence of LTBI in this study was relatively low when compared to other low and middle income countries. A systematic review by Joshi et al (2006) reported the prevalence of LTBI among HCW in low and middle income countries ranged from 33% (95% CI: 23-45) to 79% (95% CI: 75-82) [7]. The higher prevalence in the studies for this systematic review could be due to using TST for diagnosing LTBI. False positive TST results could be attributed to BCG vaccination and non tuberculous mycobacterial (NTM) infections [4]. The findings of our study were comparable to other studies which used IGRA for diagnosing LTBI. The prevalence among 2028 HCWs in Germany between December 2005 and May 2009 was 9.9% [8]. The study conducted among 332 Japanese HCW from March till April 2003 by Harada et al (2006) also had a prevalence of 9.9% [9]. Even though the prevalence is quiet similar, it should be noted that Germany and Japan are low TB-burden countries whilst Malaysia is an intermediate TB-burden country. The lower prevalence in our study could not be attributed to the inclusion of various categories of HCW who are at lower risk of exposure to TB in the workplace, as the prevalence among administrative workers was comparable with those working in the clinical side. Similar finding was also reported by Schablon et al (2010) whereby the highest prevalence of LTBI in their study was among the administration staff (17.4%) followed by ancillary nursing staff (16.7%) [8]. Does this finding indicate that there is no increased risk of LTBI among HCWs in direct contact with TB patients in Malaysia? Or are the administrative workers just as exposed to TB patients as they worked in the same facility where patients come to seek treatment? Further studies need to be conducted to ascertain these findings.

In our study, univariable analysis showed the prevalence of LTBI was significantly higher among older respondents, ever married, had a history of living in the same house with family members or friends who had TB and employed for 11 years or more. Pai et al (2005) also found an association between prevalence of LTBI with longer duration of employment [10]. They found those who were employed for 10 years or more, had a threefold increase of prevalence for LTBI compared to those who worked less than a year. Longer duration of employment was associated with longer cumulative exposure to patients with TB, hence increasing the probability of having LTBI. In our study, on multivariable analysis, duration of employment was no longer significantly associated with LTBI. This is probably because risk of infection depended primarily on the concentration of infectious droplet nuclei in the air and duration of exposure to a person with infectious TB disease [11]. Franchi et al (2007) conducted a risk assessment of an outbreak of tuberculosis in an obstetric ward in Italy, found tuberculin conversion was not related to amount of time in contact with the exposed TB patient, work shift or number of contacts with the index case. The factors that influenced tuberculin conversion in their study were brief unprotected exposure to a highly infectious case in non-ventilated small confined spaces.

The factors that were significantly associated with LTBI on multivariable analysis in our study, were being male, worked as a nurse and had history of living in the same house with a family member or friend with active TB disease. These risk factors were also reported in other studies [13,14]. Living in the same house with patients who had TB disease is a known risk factor as these individuals share the same air space and are in close contact with each other.

The agreement between QFT Git and TST in this study was 50.5% for TST with a cut off point of 10 mm and 82.1% for the cut off point of 15 mm. The κ-agreement was however poor for both cut off points. Nienhaus et al (2008) compared the agreement between IGRA and TST among those vaccinated with BCG with those who did not. They found good agreement among those who did not receive BCG vaccination and poor agreement among those who received vaccination [15]. Kang et al (2005) also noted similar findings. They attribute the poor agreement to BCG vaccination in older children and NTM infection [6]. This was probably also the case for respondents in our study. Malaysia's national immunization programme prior to 2005, involves giving primary BCG vaccination at birth and secondary vaccination at 12 years old. For those who do not have a scar or have a minimal scar, BCG is also given at 7 years of age. A study in rural India showed high agreement between the two tests even though majority of the respondents had BCG vaccination [10]. However, the researcher did mention that BCG vaccination was assumed based on the presence of a scar and there was a possibility of cross reactivity of ESAT-6 and CFP-10 with *Mycobacterium leprae *which are endemic in India. A study in German where BCG vaccination is not mandatory, it showed good correlation between IGRA and TST [15].

Limitation of this study was in determining the exposure at the workplace. Even though there was a comparison group in this study (high and low risk groups), however, some of the respondents have history of working in various departments prior to their current workplace and the timing of infection could not be determined. Other studies overcame this problem by including only respondents who had been screened previously and did not have LTBI.

## Conclusions

The prevalence of latent tuberculosis infection in Malaysia was relatively low for an intermediate TB burden country. We could not comment on the occupational risk of LTBI among health care worker compared to the general population as there are no prevalence data available for LTBI in the general population. Kappa agreement between Quantiferon TB gold in-tube and tuberculin skin test was poor. Further studies need to be conducted to ascertain the prevalence of LTBI in the general population to conclude whether there is a higher risk among HCW or not. HCWs and relevant authorities should continuously strive towards improving preventive and control measures to reduce risk of transmission of tuberculosis among HCWs.

## Competing interests

The authors declare that they have no competing interests.

## Authors' contributions

SR has made substantial contributions to the conception and design of the study, acquisition of data, analysis and interpretation of data. She has been involved in drafting the manuscript and has given final approval of the version to be published. KGR has made substantial contributions to the conception and design of the study and been involved in revising the manuscript critically for important intellectual content. He has given final approval of the version to be published. AT has been involved in analysis and interpretation of data as well as revising the manuscript. He has given final approval of the version to be published.

## Abbreviation list

AIDs: Acquired immune deficiency syndrome; BCG: Bacillus Calmette-Guérin; CFP-10: culture filtrate protein 10; CI: confidence interval; ED: Emergency Department; ELISA: enzyme-linked immunosorbent assay; ESAT-6: early secreted antigenic target 6; HCW: health care worker; HIV: human immunodeficiency virus; ICU: Intensive Care Unit; IFN-γ: interferon γ; IGRA: interferon gamma releasing assays; LTBI: latent tuberculosis infection; MA: medical assistant; MLT: medical laboratory technician; NTM: non tuberculous mycobacterial; PPD: purified protein derivative; QFT Git: Quantiferon TB Gold in-tube; SD: standard deviation; TB: tuberculosis; TBIS: Tuberculosis Information System; TST: tuberculin skin test.

## Pre-publication history

The pre-publication history for this paper can be accessed here:

http://www.biomedcentral.com/1471-2334/11/19/prepub
